# Anomalous Marginal Insertion of Umbilical Cord in Placentas of COVID-19-Affected Pregnant Mothers: A Cross-Sectional Study

**DOI:** 10.7759/cureus.33243

**Published:** 2023-01-02

**Authors:** Surekha Mullapudi Venkata, N Suneetha, Nagalla Balakrishna, K Satyanarayana, J.J. Babu Geddam, Putcha Uday Kumar

**Affiliations:** 1 Pathology and Microbiology, National Institute of Nutrition, Hyderabad, IND; 2 Obstetrics and Gynecology, Area Hospital Nampally, Hyderabad, IND; 3 Statistics, Apollo Hospitals Educational and Research Foundation (AHERF), Hyderabad, IND; 4 Pathology and Microbiology Division, Indian Council of Medical Research (ICMR) National Institute of Nutrition, Hyderabad, IND; 5 Clinical Epidemiology Division, Indian Council of Medical Research (ICMR) National Institute of Nutrition, Hyderabad, IND

**Keywords:** cord blood, marginal insertion, umbilical cord, pregnancy, covid-19

## Abstract

Aim

Study the effect of coronavirus disease-19 (COVID-19), caused by the severe acute respiratory syndrome coronavirus 2 (SARS-CoV-2), on the placenta and in turn study its effects on pregnancy and newborn outcomes.

Methods

In this cross-sectional study, which was conducted in the term pregnant women who underwent delivery, their placentas were collected after delivery along with the mothers’ blood and cord blood.

Results

Among the 212 pregnant women recruited, the prevalence of marginal cord insertion (MCI) in the placentas after delivery, was found to be 23% (n=48). Among these 48 cases (n=48) with MCI, 58.33% (n=28) were COVID-19 positive. The placentas with MCI had significantly lower minimum placental circumference (probability value/p value=0.04) and significantly longer umbilical cord (*p-value*=0.05). COVID-19 antibodies transfer from the mother to the umbilical cord (C/M antibodies ratio) was observed to be lower, albeit insignificantly. Both the weight of newborns (*p* value=0.03) and their COVID-19 antibodies levels (p-value=0.05) were observed to be significantly lower in the MCI group. Univariate analysis shows that a body mass index (BMI) ≥ 23 of the mothers was significantly associated with abnormal MCI.

Conclusion

The prevalence of MCI was observed to be high in COVID-19-affected mothers in our study. MCI was associated with lower placental size, newborn weight, lesser transfer of COVID-19 antibodies from the mother to the fetus across the umbilical cord, and lower antibody levels in the cord blood when compared to maternal blood.

## Introduction

The placenta is an important organ that connects the unborn fetus to the uterus of the mother [1]. Its main function includes the exchange of oxygen, carbon dioxide, water, electrolytes, and nutrition between the mother and the fetus [2]. The term placenta is usually discoid in shape, measuring about 15-25 cm in diameter, 3 cm in thickness, and weighing about 500-600 g [3]. The placenta is connected to the fetus with the help of an important structure called the umbilical cord (UC), which when fully developed, has a length of 40-50 cm and a diameter of 1-2 cm. The structure of the UC is known to vary at different stages of pregnancy [4,5]. The UC is an important structure that if examined as early as the first trimester of pregnancy, will increase the chances of identifying any anomalies in the early part of gestation.

Normally the UC gets inserted either in the center (central insertion) or close to the center (eccentric insertion) of the placental proper [6]. However, rarely it is found attached to the margin of the placenta when it is called marginal cord insertion (MCI) [7]. While central and eccentric insertions of the UC are more common (>90%) in term placentas and are considered normal [8], MCI and velamentous cord insertions (VCIs) (UC and its vessels get inserted into chorioamnionitic membranes instead of placenta proper) are categorized as abnormal and are known to reduce the development and alter the normal functioning of the placenta, thus adversely influencing the normal growth of the fetus and leading to abnormal birth weight and placental weight probably by modifying placental functional efficiency [8-10]. These placentas are also shown to have a sparse distribution of blood vessels in the chorionic villi [11].

The pathogenesis of this abnormal insertion of the UC is not yet deciphered. Among many theories, an important one is the theory of trophotropism, in which it is postulated that the placenta develops better in adequately perfused areas that are supplied with better blood supply and least develops in areas with low blood supply, thus indirectly causing abnormal UC insertion [8].

It is known that physiological alterations take place during pregnancy to meet increased maternal demand for oxygen, altered metabolism, pregnancy-induced anemia, and increased requirement of oxygen by the developing fetus, and these modifications tend to lower the oxygen reserve of pregnant women. This finding is very important in recent times as the world is in the grip of the deadly infection with coronavirus disease-19 (COVID-19) caused by severe acute respiratory syndrome coronavirus 2 (SARS-CoV-2) and it has affected all categories of the population, including pregnant women, who form a high-risk group due to their physiological immunodeficiency. The need for oxygen is known to increase by 21-35% as the pregnancy progresses and increased oxygen demand is also one of the symptoms of COVID-19 [12,13]. Moreover, the hypoxia induced by COVID-19 infection may adversely affect the supply of oxygen to the placenta leading to alterations in its development, blood supply, and blood vessel formation, which in turn may cause adverse effects on the developing fetus and also the newborn [14,15]. However, the effects of COVID-19 on placentation and abnormal cord insertions have not been adequately studied to date and it is important due to the additional burden of COVID-19 hypoxia on the already compromised pregnant mothers.

Different studies on the risk factors and pregnancy outcomes associated with the abnormal insertions of UC in the placenta have shown different results and a literature search by us showed a scarcity of studies dealing with anomalous cord insertion in COVID-19, which is presently a pandemic and which prompted us to take up this study [16,17].

The placenta is an important organ that connects the unborn fetus to the uterus of the mother [1]. Its main function includes the exchange of oxygen, carbon dioxide, water, electrolytes, and nutrition between the mother and the fetus [2]. The term placenta is usually discoid in shape, measuring about 15-25 cm in diameter, 3 cm in thickness, and weighing about 500-600 g [3]. The placenta is connected to the fetus with the help of an important structure called the UC, which when fully developed, has a length of 40-50 cm and a diameter of 1-2 cm. The structure of the UC is known to vary at different stages of pregnancy [4,5]. The UC is an important structure that if examined as early as the first trimester of pregnancy, will increase the chances of identifying any anomalies in the early part of gestation.

Normally the UC gets inserted either in the center (central insertion) or close to the center (eccentric insertion) of the placental proper [6]. However, rarely it is found attached to the margin of the placenta when it is called MCI [7]. While central and eccentric insertions of the UC are more common (>90%) in term placentas and are considered normal, MCI and VCIs (UC and its vessels get inserted into chorioamnionitic membranes instead of placenta proper) are categorized as abnormal and are known to reduce the development and alter the normal functioning of the placenta, thus adversely influencing the normal growth of the fetus and leading to abnormal birth weight and placental weight probably by modifying placental functional efficiency [8-10]. These placentas are also shown to have a sparse distribution of blood vessels in the chorionic villi [11].

The pathogenesis of this abnormal insertion of the UC is not yet deciphered. Among many theories, an important one is the theory of trophotropism, in which it is postulated that the placenta develops better in adequately perfused areas that are supplied with better blood supply and least develops in areas with low blood supply, thus indirectly causing abnormal UC insertion [8].

It is known that physiological alterations take place during pregnancy to meet increased maternal demand for oxygen, altered metabolism, pregnancy-induced anemia, and increased requirement of oxygen by the developing fetus, and these modifications tend to lower the oxygen reserve of pregnant women. This finding is very important in recent times as the world is in the grip of the deadly infection with COVID-19 caused by SARS-CoV-2 and it has affected all categories of the population, including pregnant women, who form a high-risk group due to their physiological immunodeficiency. The need for oxygen is known to increase by 21-35% as the pregnancy progresses and increased oxygen demand is also one of the symptoms of COVID-19 [12,13]. Moreover, the hypoxia induced by COVID-19 infection may adversely affect the supply of oxygen to the placenta leading to alterations in its development, blood supply, and blood vessel formation, which in turn may cause adverse effects on the developing fetus and also the newborn [14,15]. However, the effects of COVID-19 on placentation and abnormal cord insertions have not been adequately studied to date and it is important due to the additional burden of COVID-19 hypoxia on the already compromised pregnant mothers.

Different studies on the risk factors and pregnancy outcomes associated with the abnormal insertions of UC in the placenta have shown different results and a literature search by us showed a scarcity of studies dealing with anomalous cord insertion in COVID-19 disease, which is presently a pandemic and which prompted us to take up this study [16,17].

## Materials and methods

The present study was a cross-sectional one, which was conducted on pregnant women who were admitted to a tertiary care hospital for their delivery. The women were recruited consecutively and included women in labor. After duly explaining the study details to them and getting written informed consent from them, the mothers were enrolled in the study. The necessary due approval to conduct the study was obtained from the Ethical Committee of the Indian Council of Medical Research-National Institute of Nutrition (ICMR-NIN) with the IRB number being: 3/I/2021, before the start of the study. Recruitment was carried out between August and November 2021(when the deadly 2nd wave of COVID-19 was waning in India).

All the mothers, during the time of admission to the hospital, underwent routine screening for COVID-19 by using the COVID-19 Antigen Rapid Test Kit (Alpine Biomedicals PvT. limited, Ambala City, Haryana State, India). This was an immunochromatographic rapid assay kit for the qualitative detection of SARS-CoV-2 specific antigen in nasal swab specimens. This test kit was approved by The Indian Council of Medical Research (ICMR), Government of India. The Test Principle is as follows: Alpine One-Step COVID-19 antigen test is based on the antigen-capture immunochromatographic assay for detecting the presence of COVID-19 viral nucleoprotein antigen in the nasal swab and nasopharyngeal swab samples.

Assuming the prevalence of MCI in COVID-19 pregnancies to be 15%, with the confidence level being 95% and a 5% margin error, a sample size of 195 was calculated.

The inclusion criteria used in the study were as follows: pregnant women in the reproductive age group of 18-45 years, in their last or third trimester of pregnancy, either primiparous or multiparous, and who were not on any anti-viral medications were included in the study. The exclusion criteria were as follows: mothers suffering from acute and chronic medical conditions like renal disease, rheumatoid disease, diabetes mellitus, hypertension, abnormal pregnancies like ectopic and molar pregnancy, placentas in which the placental UC was not intact, and those with furcated and velamentous UC insertion were excluded from the study.

Once the mothers were recruited, their sociodemographic history like age, monthly income of the family, education, community, and marital status were noted. Obstetric and clinical characteristics of the mothers like gravida, parity, immunization history, previous history of stillbirths, preterm births, pregnancy-induced hypertension (PIH), abortions, fertility problems, bleeding history, history of preeclampsia, and gestational diabetes mellitus (GDM) and intake of folic acid and iron tablets were also noted.

Operational definitions

MCI was considered when the distance of insertion of the UC from the nearest placental margin was less than or equal to (≤) 2 cm and the UC was considered normal when the UC insertion was more than (>) 2 cm from the placental margin.

Collection and Processing of the Blood and Placental Samples

Blood: 5 ml of the non-fasting venous blood was collected by the medical staff, from the antecubital vein of the mothers, under strict aseptic conditions. After delivery of the newborns, 5 ml of blood was collected from the UC by the recruited staff, in the labor room, in ethylenediaminetetraacetate (EDTA) and plain vacutainers (without anticoagulants). The blood was analyzed within 6 hours of collection in the pathology laboratory.

Hematological parameters: The blood collected in EDTA was run in an automated hematoanalyzer (ADVIA 120, by Seimens, Munich and Berlin, Germany) and was analyzed for hemoglobin (Hb), packed cell volume (PCV), red blood cell count (RBC), and mean corpuscular volume (MCV).

Biochemical parameters: The serum was separated from the blood collected in plain vials, by centrifugation for 15 minutes at 1000x g at room temperature. The serum was next aliquoted and stored at -20°C until further analysis.

Anti-SARS-CoV-2 antibody quantification: This procedure was performed in the collected maternal and UC blood, by enzyme-linked immunosorbent assay (ELISA) using the COVID Kavach TM ELISA kit, which was jointly developed by the Indian Council of Medical Research-National Institute of Virology (ICMR-NIV, Pune, Maharashtra) and the Zydus Diagnostics, Ahmedabad, Gujarat, India.

**Figure 1:**
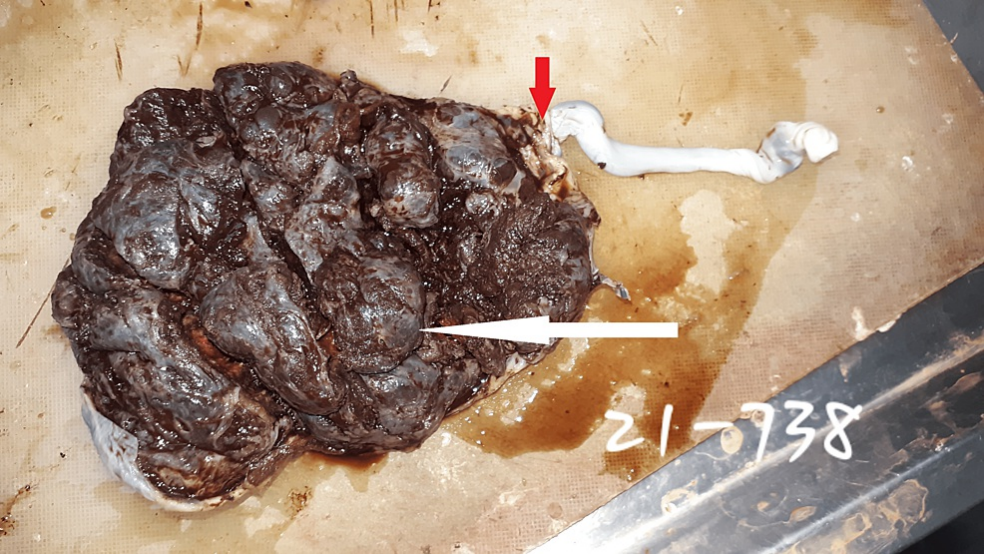
Morphology of the maternal surface of the placenta with the umbilical cord The whole placenta with the white-colored bold arrow showing the maternal surface with cotyledons and the red-colored bold arrow showing the insertion of the umbilical cord ≤ 2 cm from the margin of the placenta.

**Figure 2:**
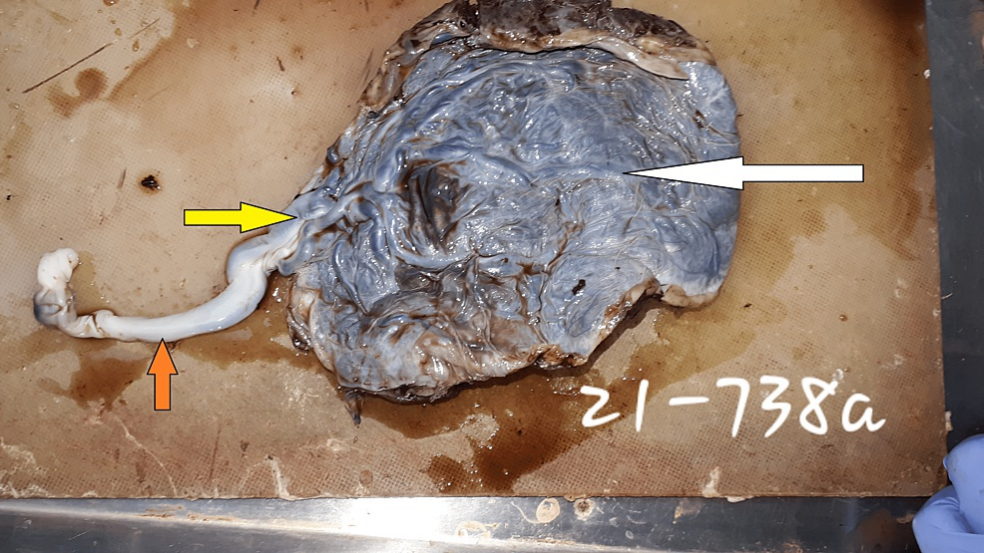
The fetal surface of the placenta with the umbilical cord attached to it Fetal surface of the whole placenta, with a bold white arrow showing the fetal surface and the bold yellow arrow pointing to the marginal insertion (≤ 2 cm from the margin of the placenta), of the umbilical cord, which is marked with an orange bold arrow.

Placenta: Immediately after delivery, the placentas were collected in 10% neutral buffered formalin and were fixed in it for 72 hours, after which their gross examination was performed. The morphological examination of the whole placenta and membranes was also done along with the UC. The length, width, and site of insertion of the UC in the placenta were noted. The distance of the UC insertion from the margins of the placentas was measured using a measuring tape. MCI was diagnosed when the distance observed between the UC insertion and the nearest margin from the placental edge was ≤ 2 cm (Figures 1-2).

Statistical analysis of the data

Mean and standard deviation (SD) values were calculated for the continuous variables and percentages were calculated for qualitative variables. The mean values of different biochemical and vital variables were compared using the Normal Curve (Z) test across UC insertion categories. The Chi-square test was applied to study the associations between UC insertion categories and socioeconomic and maternal characteristics. Regression analysis was performed to study the estimated risk of UC insertion with the significance of socio-economic status (SES), and maternal and biochemical variables as predictors. The level of significance was considered significant when the probability value (p-value) was < 0.05. Statistical Package for Social Sciences (SPSS) by the International Business Machines Corporation (IBM) version 24.0 (Chicago, USA), was used for all statistical analyses.

## Results

In our study, out of a total of 212 cases, the prevalence of MCI was found to be 23% (n=48), while the remaining 77% (n=164) showed normal cord insertion (NCI) of the UC. Among the NCI, 71% (n=151) were eccentric and 6% (n=13) were central. Among the 48 cases with MCI, 58% (n=28 cases) were COVID-19 positive and 42% (n=20) were COVID-19 negative. Table 1 shows the general characteristics of pregnant women.

**Table 1:** General characteristics of the pregnant mothers MCI: marginal cord insertion; Rs: rupees; H/o: history of; BMI: body mass index; kg/m^2^: kilogram per meter square; cm: centimeters; n(%): number (percentage), PIH: pregnancy-induced hypertension; p-value: probability value

Variables	Categories	Umbilical cord insertion			p-value
		MCI, n (%)	Normal, n (%)	Total, n (%)	
Maternal age in years	≤ 20	8 (25.8)	23 (74.2)	31 (100)	0.38
	21-25	28 (22.2)	98 (77.8)	126 (100)	
	≥ 26	12 (21.8)	43 (78.2)	55 (100)	
	Total	48 (22.6)	164 (77.4)	212 (100)	
BMI(kg/m^2^)	<18.5	0 (0)	2 (100)	2 (100)	0.95
	18.5-23	9 (14.5)	53 (85.5)	62 (100)	
	>=23	39 (26.4)	109 (73.6)	148 (100)	
	Total	48 (22.6)	164 (77.4)	212 (100)	
Education	Illiterate (No schooling)	9 (29)	22 (71)	31 (100)	0.27
	Primary & secondary school	34 (20.4)	133 (79.6)	167 (100)	
	College-educated	5 (35.7)	9 (64.3)	14 (100)	
	Total	48 (22.6)	164 (77.4)	212 (100)	
Family monthly income in Indian rupees (Rs)	<5000	14 (25)	42 (75)	56 (100)	0.15
	5000-10,000	24 (19.2)	101 (80.8)	125 (100)	
	10,000-50,000	10 (32.3)	21 (67.7)	31 (100)	
	Total	48 (22.6)	164 (77.4)	212 (100)	
Gravida	1	20 (23)	67 (77)	87 (100)	0.30
	2	17(19.8)	69 (80.2)	86 (100)	
	3	11(11)	23 (67.6)	34 (100)	
	4	0 (0)	5 (100)	5 (100)	
	Total	48 (22.6)	164 (77.4)	212 (100)	
Parity	0	18 (23.4)	59 (76.6)	77 (100)	0.74
	1	24 (23.3)	79 (76.7)	103 (100)	
	2	6 (21.4)	22 (78.6)	28 (100)	
	3	0 (0)	4 (100)	4 (100)	
	Total	48 (22.6)	164 (77.4)	212 (100)	
H/o stillbirths	Yes	0 (0)	3 (100)	3 (100)	0.46
	No	48 (23)	161 (77)	209 (100)	
	Total	48 (22.6)	164 (77.4)	212 (100)	
H/o spontaneous abortions	Yes	2 (28.6)	5 (71.4)	7 (100)	0.5
	No	46 (22.4)	159 (77.6)	205 (100)	
	Total	48 (22.6)	164 (77.4)	212 (100)	
H/o PIH	Yes	1(50)	1 (50)	2 (100)	0.4
	No	47 (22.4)	163 (77.6)	210 (100)	
	Total	48 (22.6)	164 (77.4)	212 (100)	
Number of antenatal visits	1	0 (0)	1 (100)	1 (100)	0.7
	2	0 (0)	3 (100)	3 (100)	
	3	2 (16.7)	10 (83.3)	12 (100)	
	4	4 (21.1)	15 (78.9)	19 (100)	
	5	15 (32.6)	31 (67.4)	46 (100)	
	6	23 (21.5)	84 (78.5)	107 (100)	
	7	1 (20)	4 (80)	5 (100)	
	8	3 (15.8)	16 (84.2)	19 (100)	
	Total	48 (22.6)	164 (77.4)	212 (100)	

Table 1 shows that the majority of mothers of the MCI group (n=28) and NCI group (n=98) had an age range of 21-25 years, had a BMI of ≥ 26 in MCI (n=39) and NCI (n=109) groups, were mostly primary and secondary high school educated, MCI (n=34) and NCI (n=133) and had a family income between Rs. 5000-Rs. 10,000 in MCI (n=24) and NCI (n=101) groups, respectively. The obstetric history of the participating mothers showed that in the MCI group, the majority (n=20) were primiparous with a gravida and parity of 1 (n=24), with no history of stillbirths (n=48) or spontaneous abortions (n=46), no PIH (n=47), and with six antenatal visits (n=23). The group with NCI also showed similar findings of parity 1 (n=79), no history of stillbirths (n=161), or spontaneous abortions (n=159), no PIH (n=163), and with six antenatal visits (n=84). Only the group with NCI differed from the MCI group by the majority of mothers being multiparous with gravida 2 (n=69). However, all the parameters remained statistically insignificant.

Table 2 shows the comparison of placental and newborn characteristics in the two groups with and without MCI.

**Table 2:** Comparison of placental and newborns characteristics in the two groups UC: umbilical cord; SD: standard deviation; cm: centimeters; C/M: cord by mother’s ratio; Kg: kilograms; APGAR: Appearance, Pulse, Grimace, Activity, and Respiration; COVID-19: Coronavirus disease-19; p-value: probability value *p-value: significant

Parameters	UC Insertion ≤ 2 cm from margin Mean (SD) n=48	UC Insertion >2 cm from margin Mean (SD) n=164	Overall Mean (SD)	Overall Range	p-value
Placenta					
Placental weight in grams	402.29 (80.86)	418.88 (81.79)	415.13 (81.69)	236 - 654	0.22
Maximum circumference in cm	16.62 (1.89)	17.18 (1.97)	17.06 (1.96)	13 - 26	0.08
Minimum circumference in cm	14.00 (1.67)	14.64 (1.93)	14.5 (1.89)	04 - 19	0.04*
Placental thickness in cm	2.19 (1.45)	2.03 (0.54)	2.1 (0.84)	01 - 11.5	0.24
Umbilical cord length in cm	17.21(6.26)	19.20 (6.21)	18.75 (6.27)	6.5 - 48	0.05*
C/M antibodies transfer ratio	1.07 (0.34)	1.13 (0.47)	1.12 (0.45)	0.1 - 4.2	0.38
Newborns					
Weight in kg	2.73 (0.38)	2.90 (0.47)	2.86 (0.45)	1.7 - 4.4	0.03*
Length in cm	48.81 (2.76)	49.23 (2.54)	49.12 (2.6)	40 - 58	0.37
APGAR score in 1 minute	6.71 (2.50)	6.13 (3.13)	6.26 (3.00)	0 - 8	0.24
APGAR score in 5 minutes	9.33 (0.89)	9.51 (0.53)	9.47 (0.64)	5 - 10	0.1
COVID-19 antibodies	1.97 (1.13)	2.48 (1.78)	2.36 (1.67)	0.07 - 12.37	0.05*

Table 2 shows that placentas with MCI had significantly lower minimum placental circumference (p=0.04) and a longer UC (p=0.05) when compared to the placentas with NCI. All the other parameters like placental weight, maximum placental circumference, and placental thickness, although were lower in placentas with MCI, were not statistically significant. Similarly, the COVID-19 antibodies transfer from the mother to the UC (Cord/Mother or C/M antibodies ratio) was also lower, albeit insignificantly in the MCI group. Table 2 in addition also shows the newborn parameters in the two groups. The weight of the newborns (p value=0.03) and also COVID-19 antibody levels (p=0.05) were significantly lower in the MCI group. The length of the newborns and the Appearance, Pulse, Grimace, Activity and Respiration (APGAR) score at 1 minute were also found to be lower, although without statistical significance in the MCI group while only the APGAR score at 5 minutes was higher in the same group.

Table 3 shows the comparison of clinical and hematological parameters of both mothers and their newborns in the two groups with and without presenting with MCI.

**Table 3:** Comparison of clinical and hematological parameters of both mothers and their newborns in the two groups UC: umbilical cord; SD: standard deviation; CI: confidence interval; p-value: probability value; cm: centimeters: R/R: respiratory rate; min: minute; O_2_: oxygen; %: percentage; Hb: hemoglobin; g/dl: grams per deciliter; WBC: white blood cells; µl: microliters; RBCs: red blood cells; PCV: packed cell volume; MCV: mean corpuscular volume; fl: femtoliters; IgG: immunoglobulin G: COVID-19: coronavirus disease-19 *p-value: significant

	Mothers				Newborns			
Parameter	UC insertion ≤ 2 cm from margin mean (SD) n=48	UC insertion >2 cm from margin mean (SD) n=164	p-value	95% CI	UC insertion ≤ 2 cm from margin	UC insertion >2 cm from margin	p-value	95% CI
RR/min	20.79 (9.06)	18.54 (2.31)	0.004*	18.39- 19.70	--	--	--	--
O_2_ saturation in %	98.46 (0.82)	98.58 (1.52)	0.60	98.36- 98.74	---	--	--	--
Hb (g/dl)	9.99 (2.57)	10.00 (2.07)	0.99	9.70-10.30	11.87 (2.80)	11.92 (2.25)	0.90	11.57-12.23
WBC (10^3^/µl)	10.74 (2.98)	10.39 (3.31)	0.52	10.03-10.91	8.67 (3.66)	9.91 (8.73)	0.36	8.52-10.75
RBCs (10^6^/µl)	3.89 (0.81)	3.99 (0.73)	0.45	3.87-4.07	3.56 (0.73)	3.64 (0.66)	0.47	3.52-3.72
PCV in %	31.75 (7.36)	31.95 (5.93)	0.85	31.05-32.76	36.74 (8.04)	37.13 (6.54)	0.74	36.08-38.01
MCV(fl)	80.93 (9.57)	80.43 (8.29)	0.73	79.38-81.71	103.47 (6.54)	102.23 (6.69)	0.27	101.58-103.44
COVID-19 IgG antibodies	1.93 (1.10)	2.29 (1.53)	0.13	2.02-2.41	1.97 (1.13)	2.48 (1.78)	0.06	2.14-2.59
Gestation age in weeks	39.30 (7.81)	38.17 (0.96)	0.09	37.86-39.01	--	---	--	--

Table 3 shows that respiratory rate/minute in mothers was significantly higher in mothers in the MCI group while oxygen saturation was lower in the same group, it was not statistically significant. Blood parameters like Hb, RBCs, PCV, and COVID-19 antibodies levels were lower while white blood cells (WBCs), MCV, and gestational age were higher in the mothers with MCI when compared to those with NCI, however, with no statistical significance. In the newborns, COVID-19 antibodies levels were significantly lower in the MCI group while the blood parameters like Hb, WBC, RBC, and PCV were lower while MCV was marginally higher in mothers with MCI when compared to the group with NCI, albeit with no statistical significance.

Univariate analysis performed shows in Table 4, the results of the regression analysis performed on different sociodemographic parameters with the MCI.

**Table 4:** Regression analysis of different sociodemographic parameters with the MCI MCI: marginal cord insertion; BMI: body mass index; kg/m2: kilogram per meter square; CI: confidence interval; p-value: probability value; COVID-19: coronavirus disease-19; SC: scheduled caste; ST: scheduled tribe; OBC: other backward castes; OC: open category *p-value: significant A BMI of ≥ 23 in the mothers was significantly associated with the abnormal MCI while other parameters like maternal age, education status, occupation, family income, community status, and COVID-19 status did not show any association.

Variables	Categories	Univariate analysis (p <0.05)	
		Β (95% CI)	p-value
Age of mothers in years	≤ 20	1	0.77
	21-25	1.42 (0.54-3.76)	0.47
	≥26	1.37 (0.46-4.06)	0.57
BMI of the mothers	18.5-23	1	-
	≥ 23	0.39 (0.17-0.92)	0.03*
Education status of mothers	Illiterate	1	0.19
	Primary and secondary school educated	1.75 (0.71-4.31)	0.23
	College-educated	0.64 (0.13-3.09)	0.58
Occupation of mothers	Working	1	-
	Not working	0.93 (0.12-6.87)	0.94
Monthly family income in Indian Rupees	<5000	1	0.49
	5000-10,000	1.42 (0.63-3.22)	0.40
	10,000-50,000	0.85 (0.26- 2.74)	0.78
Community	SC+ST	1	0.68
	OBC	0.48 (0.08-2.92)	0.42
	OC	0.96 (0.23-3.96)	0.96
	Others	1.09 (0.22-5.53)	0.91
COVID-19 status	Negative	1	-
	Positive	0.94 (0.44-2.00)	0.88

## Discussion

The prevalence of MCI was found to be 23% (n=48) in our study which is way higher than in other studies where the prevalence ranged from 6-15% [16-20]. Similarly in the study by Aragie and Oumer, only 6.4% of placentas showed MCI [16]. Our study was conducted when the deadly second wave of COVID-19 was slowly waning in our country. Hence, there is a greater possibility of the subjects of our study being exposed to the virus during their pregnancy period. Although the clinical history of all the participating mothers showed them to be asymptomatic during their entire pregnancy period, 53% (n=112) of them were found to be positive for COVID-19 IgG antibodies at the time of their delivery, thus pointing to their exposure to the virus during their pregnancy period. Moreover, we found in our study that among the 48 mothers who presented with MCI of their placentas, 58% (n=28) were found to be COVID-19 positive. Thus, the high incidence of MCI in our study could be possibly related to the COVID-19 infection. However, there are no studies yet to compare the results with our study.

Trophotropism is a term that refers to different variations in the site where UC gets inserted in the placenta, leading to migration of the placenta early in the pregnancy and with advancing gestation to derive better blood supply from richly vascularized areas of the placenta [5]. There is also another theory stating that “abnormal development of placenta could be due to decreased branching of the blood vessels in the chorionic villi,” thus proposing that this abnormal vasculogenesis leads to non-central insertion of the UC [21]. The above theories indicate that anomalous insertion of the UC occurs when there is a reduced blood supply to the placenta. Thrombotic events occur in up to one-third of patients with COVID-19 leading to hypoxia in the mothers [22,23]. Thus, we deciphered that COVID-19 infection-induced hypoxia in the mothers in our study could have possibly led to trophotropism and the development of abnormal placentation.

In our study, blood parameters like Hb, RBCs, and PCV values were lower while WBC and MCV values were higher in the MCI group thus indicating the presence of anemia in the group and also the high WBC levels point to COVID-19 infection in the mothers. It is known that pregnancy leads to increased oxygen demand in the mothers due to increased metabolism, anemia (very common morbidity in pregnant mothers in India), and increased fetal oxygen consumption. Increased oxygen demand is usually a common finding seen in hypoxemia and is also seen in COVID-19 infection [24]. Hypoxia which is induced due to COVID-19 may adversely affect the supply of oxygen to the placenta, thus leading to the development of complications in the delivery of infected pregnant women [14]. Moreover, studies state that hypoxia plays a significant detrimental role in the development of the fetus and also affects placentation adversely [16]. The mothers in our study were anemic and also had high WBC counts, thus pointing to infection which could be the probable cause of hypoxia and its consequences. Thus, all the above findings point to COVID-19 as the probable etiological factor responsible for the higher incidence of MCI observed in our study.

The majority of mothers (n=28) and (n=98) in both the groups (MCI and NCI, respectively) in our study belonged to a lower age range of 21-25 years. Other studies have however reported that MCI is associated with advanced maternal age and its occurrence increases with an increase in the age of the mothers [16]. However, the majority of mothers in our study belonged to a lower age group, the cause for which could be that most of the Indian population is relatively young with the latest report by the United Nations stating that youth constitute the majority of the population in India. Moreover, most of the girls in our country get married at a very young age leading to pregnancy at a very young age.

The clinical history of the mothers in our study showed that respiratory rate/minute was significantly higher in mothers with MCI along with lower oxygen saturation, thus pointing to respiratory infection as its cause and most possibly due to COVID-19.

Obstetric history of the participating mothers showed that among those mothers presenting with MCI (n=48), the majority (n=20) were primiparous with a gravida and parity of 1 (n=24). This finding of our study corroborates the finding of Ebbing et al. [25] and Aragie and Oumer [16] who similarly found that first childbirth showed an increased risk of abnormal insertion of the cord in their study. Ebbing et al. in addition also found an association between increased bleeding per vaginum in pregnancy and increased risk of MCI, which was however not observed in our study [25]. In our study, increased antenatal visits were associated with an increased incidence of MCI which probably could be due to increased chances of detection of the anomaly due to frequent prenatal check-ups.

Ebbing et al. state that anomalous UC insertion increased the risk of developing a small placenta [25]. Indeed in our study too, the placentas in the group with MCI were smaller in size with significantly lower minimum circumference when compared to placentas with normal UC insertion. Even placental weight, maximum placental circumference, and placental thickness were lower in the placentas with MCI, although with no statistical significance. The placenta and UC form undergo some adaptive changes which depend highly on some modifying factors related to the environment, specific features of mothers, and their developing fetuses [25]. Thus, in our study, the COVID-19 infection could be one of the above modifying factors causing abnormal placentation, which however needs to be corroborated by further studies.

In our study, the weight of the newborns was found to be significantly lower in the group with MCI along with their length and an APGAR score at 1 minute, thus pointing to the adverse effect of the anomalous cord insertion on the newborn outcome. A similar finding was reported by Aragie and Oumer in their study, who found a higher rate of low birth weight among the newborns in the condition of MCI and which persisted even when they adjusted for confounding factors like age of mothers, parity, hypertension history, and gestational diabetes [16].

Thus, we, for the first time in our study, attempted to study this process of maternal antibodies transfer via the UC, in the condition of anomalous cord insertion, probably caused due to COVID-19 infection. We observed that the C/M antibodies ratio was lower, albeit insignificantly, in the MCI group thus indicating reduced transfer of antibodies from the mother to the fetus through the abnormally inserted UC. Even the COVID-19 antibody levels in the blood of the newborns were found to be lower in the mothers with placental MCI when compared to the mothers having NCI. This finding thus could indicate a lesser transfer of COVID-19 antibodies from the mothers to the newborns via the UC, thus indicating the adverse effect caused by the abnormal marginal UC insertion in the placenta in our study.

A high prevalence of MCI was observed in our study when compared with other studies. MCI was related to primiparity, smaller placental size and weight, and also lower birth weight and APGAR score. A high prevalence of COVID-19 positivity was observed in the mothers with a reduced transfer of IgG antibodies from the mother to the fetus.

Limitations

The limitation of our study is that it was conducted in only one center and the results of this study, thus, cannot be extrapolated to the whole country. In addition, the study of hypoxia-related markers would have made us understand better the hypoxic effects caused by the COVID-19 infection and its relation to MCI better.

## Conclusions

Thus, we, for the first time in our study, attempted to study this process of maternal antibodies transfer via the UC, in the condition of anomalous cord insertion probably caused due to COVID-19 infection. We observed that the C/M antibodies ratio was lower, albeit insignificantly, in the MCI group thus indicating reduced transfer of antibodies from the mother to the fetus through the abnormally inserted UC. Even the COVID-19 antibody levels in the blood of the newborns were found to be lower in the mothers with placental MCI when compared to the mothers having normal insertion of the UC. This finding thus indicates a lesser transfer of COVID-19 antibodies from the mothers to the newborns via the UC, thus indicating the adverse effect caused by the abnormal marginal UC insertion in the placenta in our study.

A high prevalence of MCI was observed in our study when compared with other studies. MCI was related to primiparity, smaller placental size and weight, and also lower birth weight and APGAR score. A high prevalence of COVID-19 positivity was observed in the mothers with a reduced transfer of IgG antibodies from the mother to the fetus.
